# Clinical, Psychological, Physiological, and Technical Parameters and Their Relationship With Digital Tool Use During Cardiac Rehabilitation: Comparison and Correlation Study

**DOI:** 10.2196/57413

**Published:** 2025-04-08

**Authors:** Fabian Wiesmüller, David Haag, Mahdi Sareban, Karl Mayr, Norbert Mürzl, Michael Porodko, Christoph Puelacher, Lisa-Marie Moser, Marco Philippi, Heimo Traninger, Stefan Höfer, Josef Niebauer, Günter Schreier, Dieter Hayn

**Affiliations:** 1 Center for Health & Bioresources, AIT Austrian Institute of Technology GmbH, Reininghausstraße 13/1, Graz, 8051, Austria, 43 66478588306; 2 Ludwig Boltzmann Institute for Digital Health and Prevention, Ludwig Boltzmann Gesellschaft, Salzburg, Austria; 3 Institute of Neural Engineering, Faculty of Computer Science and Biomedical Engineering, Graz University of Technology, Graz, Austria; 4 Department of Psychology, Paris-Lodron-University of Salzburg, Salzburg, Austria; 5 University Institute of Sports Medicine, Prevention and Rehabilitation, Paracelsus Medical University, Salzburg, Austria; 6 CARDIOMED Kardiologisches Rehabilitationszentrum GmbH, Linz, Austria; 7 Institut für Präventiv- und Rehabilitationsmedizin, Cardio Vital Wels, Wels, Austria; 8 Reha Innsbruck, REHAmed-tirol GmbH, Innsbruck, Austria; 9 MedReha GmbH, Feldkirch, Austria; 10 ZARG Zentrum für ambulante Rehabilitation GmbH, Graz, Austria; 11 Department of Psychiatry II, Medizinische Universität Innsbruck, Innsbruck, Austria

**Keywords:** mHealth, telehealth, cardiac rehabilitation, wearable, adherence, health-related quality of life, intrinsic motivation, self-efficacy, health action process approach, cardiac, rehabilitation, quality of life, efficacy, psychological, physiological, digital tools, home training, monitoring, questionnaire, cardiac risk

## Abstract

**Background:**

Home and telehealth-based interventions are increasingly used in cardiac rehabilitation, a multidisciplinary model of health care. Digital tools such as wearables or digital training diaries are expected to support patients to adhere to recommended lifestyle changes, including physical exercise programs. As previously published, the EPICURE study (effect of digital tools in outpatient cardiac rehabilitation including home training) analyzed the effects of digital tools, that is, a digital training diary, adherence monitoring, and wearables, on exercise capacity during outpatient cardiac rehabilitation phase III (OUT-III) which includes an approximately 12-week home-training phase. The study encompassed 149 Austrian patients, of which 50 used digital tools.

**Objective:**

The present paper takes a deeper look into the EPICURE data to better understand the relation between the use of digital tools and various psychological, clinical, and physiological parameters, and the relation between these parameters and the improvement of exercise capacity during cardiac rehabilitation.

**Methods:**

For this work, we analyzed questionnaires concerning the patients’ cardiac rehabilitation. On all these parameters we performed 2 analyzes: (1) Comparison of the 2 groups with and without digital tools and (2) correlation with the change in the maximum workload as achieved during the exercise stress test. If data pre- and post OUT-III were available, the change in the respective parameter during OUT-III was determined and group analysis and correlation were applied on data pre OUT-III, data post OUT-III, and the change during OUT-III.

**Results:**

We found significant improvements in quality of life in both groups, with no discernible differences between patients with or without digital tools (*P*=.53). Patients with digital tools perceived significantly higher competence during cardiac rehabilitation (*P=*.05), and they anticipated higher cardiac risks if nonadherent to physical activity (*P=*.03). Although, the overall subjectively reported adherence was not significantly different in the 2 groups (*P=*.50), specific items differed. Patients with digital tools were significantly more likely to do their exercises even when they were tired (*P=*.01) and less likely to forget their exercises (*P=*.01). Concerning reasons for (non-) adherence, patients with digital tools reported significantly more often to do their exercises because they enjoyed them (*P=*.01), whereas they were significantly less likely to stop exercising when muscular pain was worse (*P=*.01*)* and to continue doing their exercises when muscular pain improved (*P=*.02). Finally, patients who reported a high level of concrete planning achieved significantly higher improvements in exercise capacity (r=0.14, *P*=.04).

**Conclusions:**

This comprehensive analysis provides valuable insights into the multifaceted impact of digital tools on outpatient cardiac rehabilitation including home training, shedding light on the importance of digital tools for increased competence and a higher risk perception during cardiac rehabilitation. In addition, the impact of digital tools on adherence and their influence on patient outcomes were assessed in the evolving landscape of digital health interventions.

## Introduction

### Rationale

Cardiovascular diseases (CVD) are the leading cause of death worldwide [1]. The burden of CVD, considering the number of disability-adjusted life years and deaths, and especially the CVD burden attributable to modifiable risk factors increase globally. Therefore, countries are recommended to implement cost-effective public health programs and interventions which target modifiable risks, promote healthy aging across the lifespan, and reduce disability and premature death due to CVD [2].

### Background

Cardiac rehabilitation is a multidisciplinary model of health care that consists of 4 phases. Phase I starts during in-hospital treatment and focusses on early mobilization. Phase II can either be performed as in-clinic or outpatient cardiac rehabilitation, depending on the availability of outpatient cardiac rehabilitation and the patient’s individual needs and preferences. Outpatient cardiac rehabilitation phase III (OUT-III) can follow both in-clinic and outpatient cardiac rehabilitation and consists of weekly visits at outpatient cardiac rehabilitation facilities. While during phase II patients are being introduced to the management of cardiovascular risk factors and subsequent lifestyle changes, phases III targets at further improving or at least maintaining results of lifestyle changes achieved during phase II [3,4]. Home- and telehealth-based interventions are increasingly being used in cardiac rehabilitation, and depending on the chosen cohort, outcomes for home-based rehabilitation may well compare with center-based cardiac rehabilitation programmes in terms of hospitalizations, quality of life (QoL), and cost [5].

The success of home- and telehealth-based cardiac rehabilitation is associated with various clinical, psychological, and physiological parameters that influence and are influenced by the patients’ adherence to the cardiac rehabilitation programme. Various studies have analyzed the effect of wearables and home-base cardiac rehabilitation on the QoL. In a recent review including 57 articles, Jones et al [6] conclude that home-based cardiac rehabilitation leads to an improved QoL and exercise capacity.

So far, there is little knowledge concerning the relationship of patients’ intrinsic motivation and digital tools during home-based cardiac rehabilitation. In a small-scale study with 23 patients, Lu et al [7] identified patients’ intrinsic motivation as a key facilitator for successful rehabilitation with remote activity sensing. The rehabilitation program, however, did not focus on cardiac patients and none of the patients were reported to have a cardiac disease. Nonetheless, considering the broader context of health behavior change, which ties in closely with multiple of the above-mentioned cardiac rehabilitation components, there is a considerable body of research supporting the importance of (intrinsic) motivation [8]. Many current psychological models of health behavior, such as the Health Action Process Approach (HAPA), place motivation and resulting intentions (motivational phase) as a prerequisites of health behavior engagement, distinguishing it from a second, volitional phase, which translates intentions into actual behavior [9]. In the motivational phase, the HAPA proposes risk perception and outcome expectancy as determinants of intentions. Consecutively, action planning, self-efficacy, and action control are proposed to affect the enactment of these health behavior intentions. Therefore, all of these are promising targets for health interventions [10-12]. Various studies analyzed self-efficacy and behavioral driving models during home-based rehabilitation [13-16], indicating that there is a huge potential for addressing psychological drivers in digital technologies used in cardiac rehabilitation. When implementing digital tools to support cardiac rehabilitation, user-friendly interfaces are crucial, since poor usability is a main barrier of digital technologies in cardiac rehabilitation, as recently highlighted by McGowan et al [17].

The multicentered EPICURE study analyzed the effect of digital tools in outpatient cardiac rehabilitation including home training. Both patient groups concluded the OUT-III according to the latest national and international recommendations for center- and home-based exercise training [18,19]. Patients aged ≥18 years who concluded OUT-III at 1 of the 5 participating institutions, who had any documented cardiovascular disease (not further specified), and who were able to provide their informed consent were eligible for the EPICURE study if they gave their written informed consent [18]. The study duration was 6 to 12 months, depending on the duration of OUT-III which is flexibly adjusted according to the underlying disease [19]. EPICURE’s primary outcomes did not support the hypothesis that the additional use of digital tools during home training lead to further improvement in the exercise capacity (P_max_) during and after OUT-III. P_max_ was assessed during exercise stress test on an ergometer under supervision of health care professionals in the cardiac rehabilitation facilities. Patients who self-reportedly used either 1 or multiple of the following technologies during OUT-III were categorized as a patient using digital tools: phone-based assessments by the attending cardiac rehabilitation facility and digital training diaries (with and without adherence monitoring done by the cardiac rehabilitation facility and with and without wearables).

This information was reported retrospectively by the patient and no protocol had been put in place to assign patient as users of digital tools (eg, minimum number of phone-based assessments) [18]. So far, the relationship between clinical, psychological, and physiological parameters as recorded in the EPICURE study had not been analyzed in detail.

### Objectives

The present paper takes a deeper look into the EPICURE data to better understand the relationship between the use of digital tools and aforementioned psychological, clinical, and physiological parameters, and the relationship between digital tools, psychological, clinical, and physiological parameters and the improvement of exercise capacity during cardiac rehabilitation. Results are discussed in relationship to the primary outcome of the EPICURE study as published in [18] and concerning their accordance with the state-of-the-art.

## Methods

### Dataset

Data were taken from the multicentric EPICURE study [18]. The dataset contained 149 patients, who were asked to answer a questionnaire concerning their cardiac rehabilitation, and to grant access to data acquired during cardiac rehabilitation. No blinding or randomization was applied since patients were recruited retrospectively. Patients were assigned to 2 groups, depending on whether they reported to have used any of the following digital tools during OUT-III: phone-based assessments, digital training diaries with and without adherence monitoring and with and without wearables. At baseline (before OUT-III), patients using digital tools were significantly younger, fitter in terms of the maximum power during ergometry (P_max_), had a lower BMI and body weight, and reported a higher QoL in all four aspects of the MacNew questionnaire prior to OUT-III [20]. More details concerning the EPICURE study population at baseline are shown in Table 1. The data in Table 1 are presented as mean (SD) and P-values were calculated with a *t* test. MacNew quality-of-life scores were assessed based on a German version of the MacNew quality-of-life questionnaire with subcategories regarding physical limitations in daily living (physical), emotional state of the patient in regard to the cardiovascular disease (emotional), effects of the cardiovascular disease on the social life and social activities (social) and an overall score over all subcategories (global) [21]. All questionnaires were recorded retrospectively post OUT-III, except for MacNew, which was recorded before and after the study duration. The structure of the analyzed data is described in the following.

**Table 1.** Study population at baseline (before outpatient cardiac rehabilitation phase III) as described in a previous study [18]*.*

Characteristics	Without digital tools	With digital tools	*P* value
**Sex, n**			
Male	107	50	—^a^
Female	30	11	—
Age (years), mean (SD)	62 (9)	55 (13)	<.001
Maximum power during ergometry (P_max_) (W), mean (SD)	142 (41)	186 (53)	<.001
BMI (kg/m²), mean (SD)	27.9 (4.7)	26.4 (4.4)	.04
Body weight (kg), mean (SD)	86 (16)	82 (13)	.13
**Blood pressure (mmHg), mean (SD)**			
Systolic	118 (11)	120 (17)	.77
Diastolic	77 (8)	75 (8)	.45
**Blood levels (mg/dL), mean (SD)**			
Glucose	105 (19)	99 (30)	.23
Cholesterol			.23
LDL	85 (32)	77 (35)	—
HDL	48 (12)	52 (12)	.04
Triglycerides	115 (53)	99 (59)	.16
**Smoking, n**			.06
Nonsmoker	28	18	—
Ex-smoker	46	23	—
Smoker	14	1	—
**MacNew score, mean (SD)**			
Physical	5.64 (0.93)	6.15 (0.81)	.01
Emotional	5.57 (0.91)	6.01 (0.8)	.02
Social	5.84 (0.91)	6.23 (0.87)	.04
Global	5.68 (0.88)	6.11 (0.76)	.02

a —: not available.

#### Quality of Life

We used the German version of the MacNew heart disease health-related QoL questionnaire [20] to assess QoL before and after OUT-III. The MacNew questionnaire was designed to evaluate how daily activities and physical, emotional, and social functioning are affected by coronary heart disease and its treatment. The questionnaire consists of 27 items concerning 3 domains (physical limitations, emotional function, and social function), with a maximum score of 7 per domain. The MacNew questionnaire is routinely assessed before and after cardiac rehabilitation by the centers participating in the EPICURE study. Therefore, unlike most other parameters, even differences of QoL pre versus post OUT-III and their relationship to the use of digital tools during OUT-III were analyzed.

#### Intrinsic Motivation

We used the German version of the Intrinsic Motivation Inventory [22] to analyze relations between intrinsic motivation, digital tools, and outcomes of cardiac rehabilitation. Intrinsic Motivation Inventory is a standardized multidimensional measurement device intended to assess participants’ subjective experience related to a target activity in laboratory experiments. The target activity was defined as physical activity during the home training phase. Intrinsic Motivation Inventory consists of 22 items concerning the categories interest and enjoyment, perceived competence, perceived choice, and felt pressure and tension.

#### Health Action Process Approach

Self-efficacy was determined based on the German version of the Self-Efficacy Scale by Jerusalem and Schwarzer [23]. In addition, we included the following parts of the HAPA described by Schwarzer [24] in the questionnaire, intention, action planning (planning), coping planning (control), outcome expectations, and risk perception. In total, 7 questions concerning intentions before OUT-III, 10 questions concerning concrete planning of physical activity during OUT-III, and 6 questions concerning control during OUT-III were provided to the participants. In addition, 1 question concerning expected health benefits of physical activity (outcome expectations) and 1 question concerning expected cardiac risk of nonadherence to physical activity (risk perception) were retrieved. All these questions were implemented in a 4-fold Likert scale.

#### Self-Reported Adherence to the Exercise Program

Patients were asked to answer 17 questions concerning their adherence to cardiac rehabilitation, which were based on the Exercise Adherence Rating Scale (EARS) [25]. Eleven out of the 17 questions concerned reasons for nonadherence, while the remaining 6 questions focused on adherence. EARS was translated to German and slightly adapted according to the study needs (refer to the self-reported adherence to the exercise program section for the detailed questions). As compared with the original EARS which applies a 5-fold Likert scale, binary responses (yes or no) were implemented.

### Statistical Analysis

The maximum mechanical power P_max_ (W) as achieved by patients during the exercise stress test at their regular assessments in the study centers pre- and post OUT-III was analyzed. The stress test was conducted through ergometry following the Austrian guidelines for exercise testing [26]. The difference ΔP_max_ (W) between P_max_ at the end of OUT-III minus P_max_ at the beginning of OUT-III was determined. If no data from the assessment before OUT-III was available, data from the assessment post phase II were taken instead since, in Austria OUT-III is expected to immediately follow phase II and, therefore, the patients P_max_ and questionnaire results are not expected to change between the end of phase II and start of OUT-III [27-29].

For each of the parameters described in Dataset section, we performed 2 analyses, (1) comparison of the 2 groups with and without digital tools and (2) correlation with ΔP_max._ Where data for both, pre- and post OUT-III, were available, the change in the respective parameter during OUT-III was determined and group analysis and correlation were applied on data pre OUT-III, data post OUT-III, and the change during OUT-III.

Normal distribution of the data was tested by the Shapiro-Wilk test. A Student *t* test was applied to test for global differences between pre- to post cardiac rehabilitation (dependent *t* test) and for differences between the groups (independent *t* test). A value of α<.05 was considered significant. Where appropriate, we included confounding variables in the ANCOVA (analysis of covariance) models as covariates, to control for theses confounders. Confounding variables were all variables which significantly differed between patients without versus patients with digital tools determined by *t* tests. Changes pre- versus post cardiac rehabilitation were analyzed with boxplots and 2-tailed *t* tests based on matched pairs. The correlation coefficients “*r*” were calculated using Pearson’s correlation coefficient and the significance of the influence was calculated using ANCOVA. If not stated otherwise, the cofounders considered in the ANCOVA were the age, P_max_, body mass index, and the global MacNew score, all measured at the start of OUT-III.

### Ethical Considerations

The EPICURE study protocol was approved by the ethics committee of Upper Austria (vote nr. 1165/2019) and registered at ClincalTrials.gov (Identifier: NCT04458727). All analysis performed during this work are covered by this ethics approval. All participants received oral and written information before the study entry and provided written informed consent to participate in this study. This informed consent also covered the analysis of secondary study objectives performed in this paper. All data were recorded in a pseudonymized way and anonymity was ensured for all participants during presentation of the findings. Patients did not receive any financial incentives for participating in the study.

## Results

Table 2 shows the mean (SD) of the quality-of-life as reported by the MacNew questionnaire (mean per subcategory, 1…strongly disagree, 7… strongly agree). The number of data points available for each analysis is shown in brackets. Subcategories (physical, emotional, and social) scores and an overall score aggregated over all categories (global), pre- and post OUT-III, as well as the difference between pre- and post OUT-III are shown (*P* value pre vs post). Differences between the groups with and without digital tools are provided (*P* value between groups). As illustrated in Table 2, the QoL according to the MacNew score aggregated over all subcategories (global), significantly improved in patients without (*P=*.01) as well as patients with digital tools (*P=*.01). A significant increase in the physical subquestionnaire (physical) has been identified for the group without (*P*=.01) and with digital tools (*P=*.04). The group with digital tools showed a significant improvement concerning the emotional QoL subsection (*P=*.01) while the group without digital tools achieved a significant increase in the social QoL score (*P=*.01).

**Table 2.** Mean (SD) of the quality of life as reported by the MacNew questionnaire (mean per subcategory, 1…strongly disagree, 7… strongly agree).

Subcategories		Without digital tools		With digital tools		*P* value between groups
		Mean (SD)	Number of data points, n	Mean (SD)	Number of data points, n	
**Physical**						
Pre		5.64 (0.93)	74	6.15 (0.81)	32	.02
Post		5.92 (0.82)	63	6.38 (0.8)	13	.04
*P* value pre versus post		.01	62	.04	13	.21
**Emotional**						
Pre		5.57 (0.91)	74	6.01 (0.8)	32	.01
Post		5.71 (0.95)	63	6.4 (0.7)	13	.01
*P* value pre versus post		.15	62	.01	13	.94
**Social**						
Pre		5.84 (0.91)	74	6.23 (0.87)	32	.07
Post		6.11 (0.84)	63	6.42 (0.79)	13	.06
*P* value pre versus post		.01	62	.84	13	.78
**Global**						
Pre		5.68 (0.88)	74	6.11 (0.7)	32	.03
Post		5.91 (0.85)	63	6.39 (0.73)	13	.02
*P* value pre versus post		.01	62	.01	13	.53

**Figure 1.**
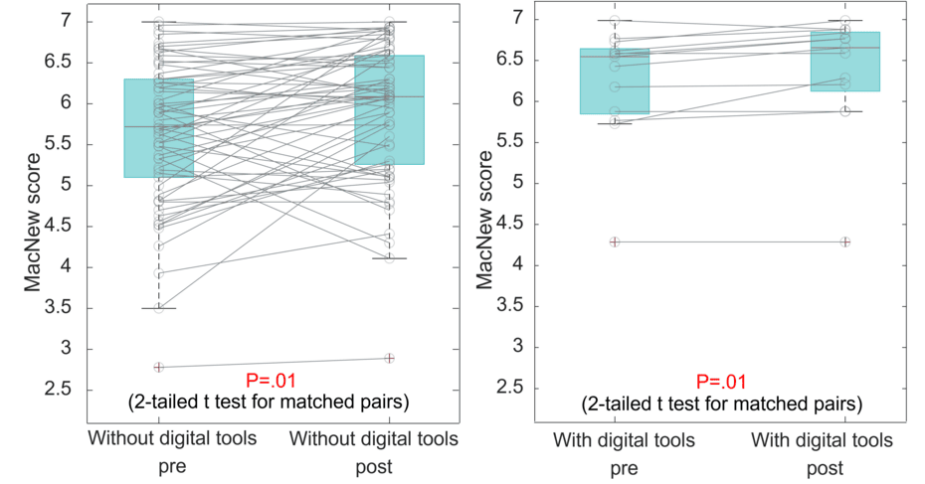
Quality of life according to the global MacNew score pre- versus post outpatient cardiac rehabilitation phase III for patients without digital tools (left) and with digital tools (right), illustrated as boxplots with matched pairs.

The change of the global QoL pre- versus post OUT-III for both groups is illustrated in (Figure 1). Although both groups improved, no significant differences in the improvement between the 2 groups were found according to the ANCOVA analysis, neither for the global MacNew score nor for any of the subcategories. Markers in pre- and post which are connected by lines correspond to one and the same patient.

As shown in Table 3, there were no correlations between changes in QoL scores and ΔP_max_.

**Table 3.** Pearson’s correlation coefficient *r* (*P* value calculated by analysis of covariance) between changes of quality of life in subcategories physical, emotional, and social as well as the global overall score as reported by the MacNew questionnaire and the change in exercise capacity ΔP_max_.

Subcategories	Correlations with ΔP_max_	
	*r* value	*P* value
ΔPhysical	0.2	.16
ΔEmotional	0.09	.84
ΔSocial	0.18	.28
ΔGlobal	0.19	.20

### Intrinsic Motivation Scale

Table 4 shows results per subsection of the Intrinsic Motivation Scale (IMS) as well as the aggregated score over all 4 categories. A significant difference in the item group concerning perceived competence was found between patients with versus without digital tools (*P=*.05), however, no significant correlation with ΔP_max_ was identified (*P=*.57). In addition, the IMS score for interest and enjoyment and the total IMS score showed borderline significant differences between the groups. In all significant and borderline significant cases, the group with digital tools achieved higher scores.

**Table 4.** Mean (SD) of the Intrinsic Motivation Scale (1…strongly disagree, 5…strongly agree) and differences between the groups and Pearson’s correlation coefficient *r* (*P* value calculated by analysis of covariance) between the Intrinsic Motivation Scale and change in exercise capacity ΔP_max_.

Subcategories	Influence of the group			Correlation with ΔP_max_	
	Without digital tools, mean (SD)	With digital tools, mean (SD)	*P* values	*r* values	*P* values
Interest and enjoyment	2.58 (0.83)	3.16 (0.68)	.08	0.27	.41
Perceived choice	3.15 (0.78)	3.49 (0.69)	.50	0.20	.39
Perceived competence	2.58 (0.78)	3.24 (0.78)	.05	0.23	.57
Felt pressure and tension	2.80 (0.84)	3.10 (0.91)	.66	0.06	.31
Total	2.77 (0.62)	3.25 (0.57)	.09	0.25	.74

### Health Action Process Approach

As summarized in Table 5, we did not identify a significant difference between the patients without and with digital tools concerning the self-efficacy (*P*=.95). In addition, there was no significant correlation between self-efficacy and the change in exercise capacity ΔP_max_ (*P=*.16). We did not identify any significant differences between patients without and with digital tools regarding the intention, control, and planning scores. However, the planning score was significantly correlated with ΔP_max_ (*P=*.04, *r=*0.14). Table 5 also shows results concerning the patients’ expected health benefit of physical activity (outcome expectations) and cardiac risk of nonadherence to physical activity (risk perception). Borderline significance (*P=*.06) was found between outcome expectations and ΔP_max_. Patients with digital tools reported to expect significantly higher cardiac risks, if they were adherent to physical activity (*P=*.03).

**Table 5.** Mean (SD) of health action process approach scores (Self-efficacy: sum of 10 items, each item 1…strongly disagree, 4…strongly agree; all other parameters: mean per category, 1…strongly disagree, 4…strongly agree) per group and difference between group including *P* value and Pearson’s correlation coefficient *r* (*P* value calculated by analysis of covariance) between the scores and change in exercise capacity ΔP_max_ during OUT-III.

Subcategories	Influence of the group			Correlation with ΔP_max_	
	Without digital tools, mean (SD)	With digital tools, mean (SD)	*P* values	*r* values	*P* values
Self-efficacy	31.80 (3.91)	32.20 (5.55)	.95	0.08	.16
Intention	3.33 (0.49)	3.46 (0.5)	.92	0.11	.36
Control	3.22 (0.59)	3.51 (0.52)	.21	0.17	.13
Planning	2.90 (0.57)	3.15 (0.62)	.10	0.14	.04
Outcome expectations	3.75 (0.54)	3.88 (0.33)	.82	0.13	.06
Risk perception	2.95 (0.83)	3.31 (0.85)	.028	0.02	.57

### Self-Reported Adherence to the Exercise Program

**Figure 2.**
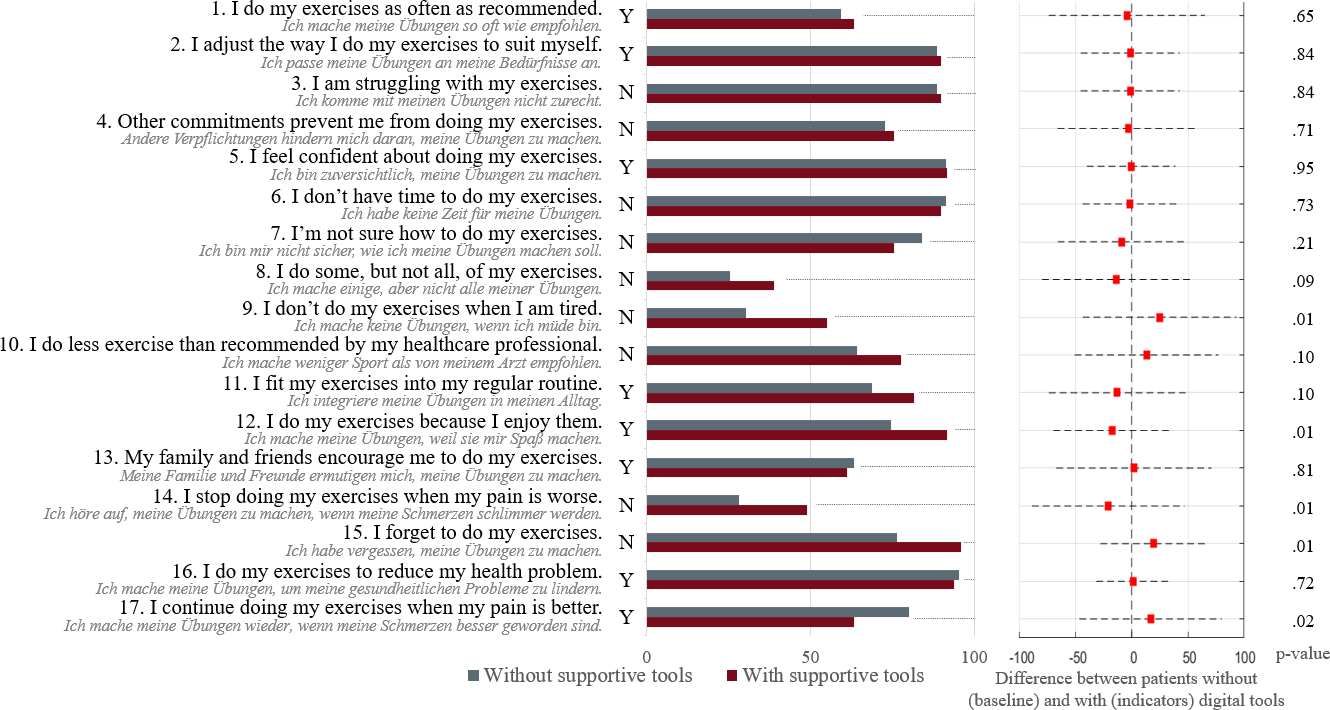
Results of the subjective adherence questionnaire per group with the original questions in German (gray) and the English translation (black).

As shown in Figure 2, patients with versus without digital tools significantly differed in five out of 17 EARs related questions. The left of Figure 2 shows the percentage of patients per group, answering with Yes or No as indicated left to the bars. Yes or No are aligned alternatively, depending on positive or negative phrasings of the respective questions, so that end-to-end bars relate to positive behavior. The right of Figure 2 shows the difference of the score per item between patients with versus without digital tools, including the corresponding *P* value.

Concerning adherence-related items, patients without digital tools reported to do their exercises significantly less often when tired (item 9). Patients with digital tools reported to forget to do their exercises significantly less often (item 15).

Concerning reasons for nonadherence, item 12 ("I do my exercises because I enjoy them") was significantly more important in patients with digital tools. Pain-related items were more important in the group without digital tools (item 14 and item 17), although high scores of item 14 represent low adherence, while high scores for item 17 relate to high adherence.

The overall EARs score showed no significant difference between the patients without (11.80 [2.7]) and patients with (12.80 [2.79]) digital tools (*P*=.5). No significant correlation between the overall score and ΔP_max_ was found (*P*=.21, *r*=0.07). Also, the adherence specific score as calculated by items 1, 3, 8, 10, 11, and 15 resulted in no significant difference between patients without (3.79 [1.71]) and patients with (4.38 [1.58]) digital tools (*P*=.57). No correlation between the adherence specific score and ΔP_max_ was found (*P*=.27, *r*=0.01). These scores were calculated with the sum of the selected items (0…no, 1…yes).

## Discussion

### Principal Findings

In this work, we analyzed the secondary hypotheses of the EPICURE study (primary outcomes have been published previously [18]), that is, the effects of digital tools in OUT-III. Our main findings are that, according to the MacNew questionnaire, QoL improved significantly during OUT-III, with similar improvement in patients without and with digital tools indicating that the use of digital tools did not significantly influence the increase of QoL during OUT-III. Furthermore, we found that patients with and without digital tools significantly differed in the following aspects:

1. Patients using digital tools perceived a significantly higher competence during cardiac rehabilitation compared with patients without digital tools, suggesting that either digital tools improve the competence or patients feeling more competent tend to use digital tools more often.
2. Patients with digital tools reported significantly more often to expect an increased risk for future cardiac events if they were not adequately physically active in the future as determined by the health action process approach.
3. Patients with digital tools were significantly less likely “not to do their exercises when they are tired” and to “forget to do their exercises.” Concerning reasons for (non) adherence, patients with digital tools significantly more often reported to do their exercises because they enjoyed them. In addition, patients with digital tools were significantly less likely to stop exercising when pain got worse and to continue doing their exercises when there was a relieve in pain. However, the subjectively reported overall adherence was not significantly different in the 2 groups.
4. Patients who reported a high level of concrete planning to perform exercise training during cardiac rehabilitation achieved significantly higher improvements in ΔP_max_.

### Comparison With Previous Work

The previously published EPICURE paper [18] summarized that, overall, exercise capacity improved both in patients with and without digital tools. The exercise capacity was measured during ergometry in 5 different Austrian rehabilitation centres. No data concerning the inter-rater reliability between the centres was available for analysis. When comparing changes with *t* tests, patients with digital tools improved significantly more than patients without digital tools. However, this change did no longer reach statistical significance when correcting for confounders with an ANCOVA analysis. In this paper, wherever suitable, *P* values were based on ANCOVA corrected for confounders.

#### Quality of Life

Jones et al [6] reported that digital tools resulted in a significantly higher improvement of QoL for the home-based compared with the center-based cardiac rehabilitation group. Although we identified a significant improvement in both groups, no significant difference in the improvement of the QoL between patients with or without digital tools was found. In both groups, QoL was already high before OUT-III, with a mean of 5.68 (without) and 6.11 (with digital tools) out of 7 possible points. These high QoL scores before OUT-III in combination with a small sample size of patients with MacNew data pre- and post OUT-III, especially in the group with digital tools (n=13), might explain the difference between Jones et al [6] and our results. Jones et al [6] also analyzed multiple studies of which the majority (44 out of 55) were randomized controlled trials, which differed from the retrospective EPICURE setting.

#### Intrinsic Motivation

The higher IMS scores in the group with digital tools are in line with Lu et al [7], who identified patients’ intrinsic motivation as a key facilitator for successful rehabilitation of chronically ill patients and patients on the verge of being chronically ill with remote activity sensing. Similarly to the EPICURE study, Lu et al [7] conducted retrospective analysis on a smaller patient cohort of 23 patients which combined a mixed-methods approach, of retrospective questionnaires and interviews with real time activity sensing using wearable devices. Our results indicate that digital tools can also help to motivate cardiac patients, without exposing patients to additional distress. However, since IMS was asked retrospectively, patients who knew that they were adherent may have been influenced by this knowledge when retrospectively evaluating their motivation before the program. Therefore, and since there is only little literature on this topic so far, prospective studies might be indicated to get a deeper insight into the relationship between intrinsic motivation and digital tools during cardiac rehabilitation.

#### Health Action Process Approach

Unlike a previous study by Salah Eldin Saad et al [30] which showed significant differences in self-efficacy, our study indicates that self-efficacy neither differed between patients with and without digital tools, nor was there a significant correlation with the achieved change in exercise capacity. In part, this result can be explained by the retrospective nature of the EPICURE study compared to a prospective setting of Salah Eldin Saad et al [30] who divided the patients equally before the cardiac rehabilitation. However, the relationship between self-efficacy, digital tools and outcome of cardiac rehabilitation might further be studied in the future. Additionally, the intervention of Salah Eldin Saad et al [30]was based on regular assessments of patients’ progress and provided cardiac rehabilitation relevant information weekly to patients using digital tools, ensuring a personalized and tailored rehabilitation process. Even though personal phone-based assessments were also part of the EPICURE study, these were not structured and routinely performed [30].

The change in exercise capacity ΔP_max_ correlated significantly with the level of planning, as retrieved from ten items in a respective questionnaire (Table 5). No significant difference between patients with and without digital tools was identified. In addition, there was no significant difference or correlation concerning the intention and control dimension of the questionnaire. As Scholz et al [10] suggested in their 5-week longitudinal on-line study with 354 patients, planning predicts behavior when intention scores are high. The study by Scholz et al [10] focused more thoroughly on the planning stage the EPICURE study since patients were first encouraged to articulate their intentions regarding physical activity and subsequently outlined specific plans that detail the context and timing of their intended activities [10]. Even though this process was not implemented to this extent in EPICURE, the influence of the planning on the execution was confirmed by our findings that showed a significant correlation between the planning score and ΔP_max_.

The outcome expectation showed a marginally significant influence on ΔP_max_, therefore, it might be beneficial to strengthen the patients’ expectation during OUT-III. Even though the risk perception did not influence the outcome ΔP_max_ we identified a significant difference between patients with and without digital tools. Since patients with digital tools had a higher score on the risk question this might be an indicator that patients who are more aware of their risk are more willing to participate in home-based cardiac rehabilitation.

#### Self-Reported Adherence to the Exercise Program

As previously discussed in a meta-analysis by Li et al [31] telehealth in a cardiac rehabilitation setting, which is based on digital tools (a combination of instant communication tools, smartphone applications, and wearable devices) improves exercise capacity. Home-based and community-based telehealth cardiac rehabilitation in the secondary phase were eligible interventions. These were compared with a combination of traditional cardiac rehabilitation or usual care [31]. This corresponds to our findings, since results from the exercise adherence questionnaire revealed that the use of digital tools in cardiac rehabilitation is associated with higher exercise adherence and to support patients to do their exercises even when they are tired. In addition, patients with digital tools reported more often to do their exercises because they enjoy them. This is in line with the results from the IMS questionnaire, which also showed higher interest and enjoyment scores in the group with digital tools. Differences in the IMS scores did, however, not reach statistical significance. Since the data were analyzed retrospectively, people who decided to use digital tools might have been more motivated in general and, therefore, better in adhering to their exercise plans.

Significant differences in the subjective adherence scores related to pain are expected to be caused by different baseline characteristics concerning pain the 2 groups. However, since pain itself was not recorded in our study, these results might require further analyses in the future.

### Limitations

Most questionnaire data were recorded at the time of study entry of the patients, that is, post OUT-III. Therefore, except for those questionnaires that were routinely recorded during cardiac rehabilitation (eg, MacNew questionnaire prior to OUT-III), all questions dealing with data before the end of OUT-III need to be interpreted with care, since the answers might have been influenced by the patients’ experiences during OUT-III and different answers might have been given in a prospective setting.

During sample number calculations in the study preparation phase, we assumed that approx. A total of 50% of patients would report to have performed cardiac rehabilitation with and without digital tools, respectively. However, questionnaire data revealed that approx. One third did and 2 thirds did not use digital tools, which reduced the power of our analyses. Since especially the number of patients using digital tools was rather low, additional studies with larger sample size may be indicated. In addition, not all patients had valid data at the start of OUT-III. For these patients data from the end of phase II was used with the assumption that the relevant physiological and psychological characteristics did not change in the short period between phase II and OUT-III. For future work, a prospective study is warranted that randomizes patients to either a group with or without digital tools, preferably including stratification on those baseline parameters that were found to have significant influence on cardiac rehabilitation outcomes, that is, age, P_max_, BMI, body weight, and QoL.

### Conclusion

We conclude that digital tools used during OUT-III including home-training can increase perceived confidence and improve joy during cardiac rehabilitation. In addition, digital tools can help to not forget exercises and to do the exercises even when tired. Concrete planning is correlated with better improvement in exercise capacity and should be fostered during OUT-III.

## Abbreviations

ANCOVA: analysis of covariance
CVD: cardiovascular diseases
EARS: Exercise Adherence Rating Scale
HAPA: Health Action Process Approach
IMS: Intrinsic Motivation Scale
OUT-III: outpatient cardiac rehabilitation phase III
QoL: quality of life
